# Next‐generation SNPs from lignin biosynthesis gene and transcription factors reveal a hidden teak (*Tectona grandis* L. f.) lineage: implications for plantation resilience and forest management

**DOI:** 10.3389/fgene.2026.1842536

**Published:** 2026-08-07

**Authors:** Nuzhat Bano, Naseer Mohammad, Shamim Akhtar Ansari

**Affiliations:** 1 Forest Physiology and Molecular Biology Division, ICFRE–Institute of Forest Productivity, Ranchi, India; 2 Genetics and Tree Improvement Division, ICFRE–Tropical Forest Research Institute, Jabalpur, India; 3 Integral University, Lucknow, India

**Keywords:** AMOVA, cryptic subpopulation, inbreeding coefficient, minor allele frequency, phylogenetic tree, principal coordinate analysis

## Abstract

**Introduction:**

Teak (*Tectona grandis* L. f.) is one of the world's most valuable tropical timber‐producing tree species, and understanding its genetic diversity and population structure is fundamental for sustainable breeding, conservation, and plantation management. In the investigation, we employed next‐generation single nucleotide polymorphism (SNPs) markers to investigate the genetic diversity and population structure of Indian teak.

**Methods:**

A total of 98 SNPs derived from three lignin biosynthesis gene and transcription factors were utilized to estimate the genetic diversity and population structure of teak collected from 10 agro climatic states and maintained at NTGB, Chandrapur, India.

**Results:**

Among the studied populations, the Odisha teak population exhibited the highest allelic richness (P% = 88.78%, Na = 1.92, I = 0.32), whereas the Kerala teak population displayed the highest genetic diversity indices (Na (rar) = 1.16, Ne = 1.33, Ho = 0.15, and uHe = 0.20), identifying it as a key hotspot of genetic variation. Structural analysis based on the Pritchard model revealed three gene pools in Indian teak, providing novel genetic evidence that extends beyond the previously recognized two‐tier population framework comprising southern and central Indian lineages. The third, previously undetected, hidden gene pool may represent an introduced lineage and is hypothesized to have originated from Myanmar. The high inbreeding coefficient of the third gene pool (F_IS_ = 0.47−0.57) is consistent with the hypothesis that this lineage may have experienced population bottlenecks, genetic isolation, and restricted gene flow. AMOVA revealed that the majority of the genetic variation was captured within the subpopulations/genetic clusters (62.29%), and the remainder was among the subpopulations/genetic clusters (35.73%), with significant genetic differentiation (F_ST_ = 0.38, P < 0.001).

**Discussion:**

These findings reveal a previously unrecognised genetic lineage in Indian teak, providing new insights into its population structure. Accurate documentation of native and introduced gene pools will support the identification of genetic diversity hotspots. The majority of Indian teak belongs to central Indian teak lineage; greater utilization of genetically diverse southern Indian teak could broaden the genetic base and enhance long‐term sustainability.

## Introduction

Teak (*Tectona grandis* L. f.) is a large, deciduous, tropical hardwood tree from the family Lamiaceae (2n = 36). It is renowned for its premium quality timber (Gill et al., 1983). The exceptional demand for teak timber is attributed to its remarkable strength, durability, and aesthetic appeal, which are characterized by golden-colored wood rays and natural resistance to termites (Dungani et al., 2012), fungi (Rachana, 2009), rot, and decay (Sumthong et al., 2008). Teak wood is used for making high-grade furniture, boat decks, doors, decorative items, seals and other wooden products (Williams et al., 2001). The species is extensively cultivated across tropical and subtropical regions because of its high economic value and elite quality timber. Teak exhibits a discontinuous natural distribution influenced by environmental factors such as rainfall, temperature, soil conditions, and light availability (Troup, 1921). The species is naturally distributed across India, Myanmar, Thailand, and Laos, with India representing a major center of teak diversity. Although Myanmar was initially considered the probable center of origin because of its extensive natural forests (Méniaud, 1930), later population genetic studies suggested India as a probable center of origin due to its higher genetic diversity and broader gene pool (Hansen et al., 2015).

In India, natural teak forests are mainly distributed across central and southern regions, including Madhya Pradesh, Kerala, Karnataka, Maharashtra, Tamil Nadu, and Andhra Pradesh. At present, teak plantations are extensively found in Bihar, West Bengal, Haryana, Assam, Meghalaya, and Uttar Pradesh (Chakrabarti and Gaharwar, 1995). The number of teak plantations in India is approximately 2.51 million ha, with 1,255 ha of clonal seed orchards (Katwal, 2005), 393 ha of seedling seed orchards (Rao, 2005) and 5,541 ha of seed production area (Forest Survey of India, 2001). The high demand for teak timber has led to its widespread use across various Indian states. However, the genetic origin and diversity of planting materials used in many early plantations remain poorly documented. Inadequate documentation of planting materials has limited the understanding of genetic relationships and diversity patterns among teak populations in India. The knowledge of genetic pool and natural genetic variability is essential for genetic improvement programs, as it enables accurate estimation of heritability, assessment of genetic gain, and effective management of genetic resources. Genetically diverse gene pools also enhance adaptability and resilience to climatic and biotic or abiotic stresses (Jump et al., 2009; Vinceti et al., 2020).

A variety of molecular markers have been employed for genome analysis and genetic diversity studies in tree species. Second-generation PCR-based markers, including RAPD, AFLP, SSR, and ISSR, have been extensively used to assess genetic diversity and population structure in natural and planted teak populations (Narayanan et al., 2007; Nicodemus et al., 2005; Watanabe et al., 2004; Shrestha et al., 2005; Balasundaran et al., 2010; Vaishnav et al., 2014; Alcantara and Veasey, 2013; Fofana et al., 2009; Verhaegen et al., 2010; Win et al., 2015b; Hansen et al., 2015; Ansari et al., 2012).

More recently, third-generation single-nucleotide polymorphism (SNP) markers have gained importance because of their high reproducibility, abundance, and efficiency in detecting genetic diversity and population structure (Brookes, 1999; Gupta et al., 2008; Würschum et al., 2013). Advancements in teak genomics, including draft and chromosome-scale genome assemblies, have considerably improved the understanding of genome organization and gene content in teak (Yasodha et al., 2018; Sahu et al., 2023). In addition, genome-wide gene family characterization studies have also expanded the available genomic resources in teak (Alhindi et al., 2026). However, SNP-based diversity studies in Indian teak remain limited. Previous studies based on genome-wide SNPs conducted in Ghana, Brazil and Indonesia included only 1-4 Indian genotypes (Wanders et al., 2021; Dos Anjos et al., 2023) and 36 genotypes belonging to Malabar, Central provenance and Godavari region only (Onuma et al., 2025). Callister et al. (2024) utilized genome-wide SNP markers for genomic prediction and identification of loci associated with growth and wood quality traits. In addition, Bano et al. (2024) employed candidate gene-based SNP markers for association analysis of wood quality traits in teak. Among the limited SNP-based studies available in Indian teak, Patturaj et al. (2025) performed one of the most comprehensive investigations employing whole-genome re-sequencing and high-density genome-wide SNP markers to analyze population structure, linkage disequilibrium, and wood trait associations in 132 teak genotypes conserved in the National Teak Germplasm Bank. However, the study primarily focused on population structure and trait associations. The detailed assessment of state wise genetic diversity, genetic differentiation and population structure of Indian teak using high resolution SNP markers remains to be explored, particularly for understanding whether planted teak populations are of indigenous origin or derived from introduced germplasm.

We hypothesized that hidden (cryptic) population structure exists within Indian teak populations, resulting in significant genetic differentiation, and that some admixed populations may reflect introduced germplasm. Therefore, the present study aimed to utilize candidate gene-derived SNP markers from gene CAD1 and transcription factors MYB1 and MYB2 (Bano et al., 2024) to: (i) assess genetic diversity and population structure among Indian teak populations, (ii) evaluate genetic differentiation across major teak populations, and (iii) identify possible indigenous and exotic cryptic gene pools within Indian teak germplasm. The findings of the study are expected to contribute to effective germplasm management, conservation, and teak improvement programs.

## Materials and methods

### Plant sampling

**FIGURE 1:**
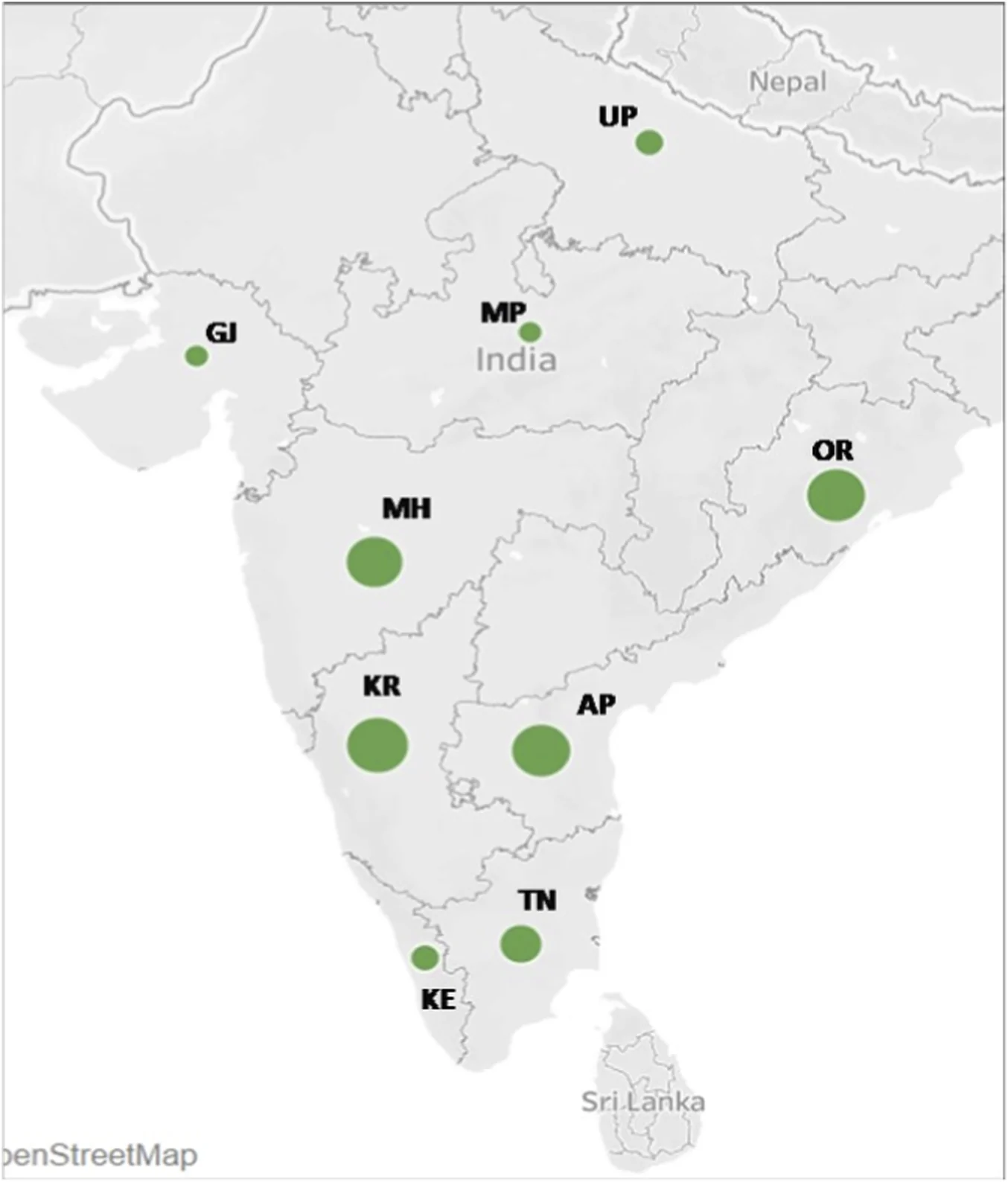
Map of India showing the sampling location of teak; the green circles represent the sample size per state.

The National Teak Germplasm Bank (NTGB), Chandrapur, Maharashtra, India, assembled a diverse collection of bud-grafted plus trees (PTs) of teak, which were selected from natural teak forests and plantations across various Indian states. A total of 147 teak genotypes, representing ten different ecological regions, were included in this study. The NTGB samples included 29 genotypes from Karnataka (KR), 13 from Tamil Nadu (TN), and 6 from Kerala (KE), representing high-rainfall states and 26 genotypes from Odisha (OR), 24 from Maharashtra (MH), 26 from Andhra Pradesh (AP), 6 from Uttar Pradesh (UP) and 4 genotypes each from Gujarat (GJ) and Madhya Pradesh (MP), representing average to optimum rainfall states. NTGB has assembled some genotypes with unknown locations of origin and those genotypes were categorized as “All India (AL_IN)” (Table 1; Figure 1). The sampling states possessing unequal sampling size need to be interpreted with caution for estimating true genetic diversity and population structure of Indian teak.

**TABLE 1:** List of 147 teak genotypes and their state-wise geo-climatic characteristics.

States	Code	Number of genotypes collected	Latitude (N)	Longitude (E)	Elevation (m)	Average annual rainfall (mm)
All India	AL_IN	9	20.59°	78.96°	247	1,083
Andhra Pradesh	AP	26	15.91°	79.74°	99	1,094
Gujarat	GJ	4	22.26°	71.19°	293	1,107
Kerala	KE	6	10.85°	76.27°	49	3,055
Karnataka	KR	29	15.32°	75.71°	651	1,248
Maharashtra	MH	24	19.75°	75.71°	505	939
Madhya Pradesh	MP	4	22.97°	78.66°	334	1,017
Odhisa	OR	26	20.95°	85.10°	123	1,489
Tamil Nadu	TN	13	11.13°	78.66°	138	998
Uttar Pradesh	UP	6	26.85°	80.95°	117	1,025
Total	​	147	​	​	​	​

The genotypes assembled at, and sampled from National Teak Germplasm Bank, Chandrapur, Maharashtra, India.

### DNA extraction and quantification

For the extraction of DNA from fresh young leaves, shoot cuttings of NTGB plus trees were collected and surface sterilized with 0.1% aqueous mercuric chloride solution for 5 min, followed by basal treatment with 5 mM IBA for 24 h. The treated cuttings, which were sealed at the top cut ends with molten paraffin wax, were planted and sprouted in the raised sand beds of the mist chamber maintained at ambient temperature and 70%–80% RH. The sprouted leaves were harvested, and their genomic DNA was extracted via a modified CTAB method of Narayanan et al. (2007). The purity and quantity of the extracted DNA were measured via biophotometer plus (Eppendorf, @613200008), and the quality and integrity of the extracted DNA were verified via 1% horizontal agarose gel electrophoresis (Tarsons @7,050).

### SNP genotyping through a resequencing approach

The lignin biosynthesis gene *CAD1* and the transcription factors *MYB1* and *MYB2* were amplified from 147 teak genotypes. The amplified products were directly sequenced through the Sanger sequencing (di-deoxy chain termination) method in both forward and reverse orientations. The base call quality of each sequencing reads was assessed via FinchTV, and sequence alignment was performed via BioEdit version 7.0.5.3 and ClustalX version 2.1. Single nucleotide polymorphisms (SNPs/indels) from the sequencing reads were identified via the variant caller NovoSNP software v3.0.1. A total of 123 SNPs/indels were identified, of which, loci with MAF ≤1.1% and missing data ≤7.3% were discarded from the analysis and 98 high-quality SNPs/indels were retained for downstream analysis. Since the SNPs were derived from a limited set of candidate genes, the possibility of locus-specific bias can be a limitation of this study. Further, the low MAF threshold (≥1.1%) was selected considering the candidate gene re-sequencing approach, which typically yields lower frequency variants. The detailed SNP/Indel discovery procedures are described in Bano et al. (2024). The SNP variants used in this study were deposited in the European Variation Archive (EVA) (Cezard et al., 2022) under accession number PRJEB95880 (https://www.ebi.ac.uk/ena/browser/view/PRJEB95880).

## Data analysis

### Genetic diversity

Single-nucleotide polymorphism (SNP) data were numerically coded as 1/1 for the homozygous major allele, 2/2 for the homozygous minor allele, 1/2 for heterozygous calls and ?/? Or −1/-1 for missing data as required by the respective software tools. Since, SNPs are bi-allelic in nature, locus wise coding of major allele (1/1), minor allele (2/2) and heterozygous locus (1/2) preserved complete allelic information without loss of genetic signal for downstream analyses. The locuswise minor allele frequency, gene diversity and polymorphic information content (PIC) were estimated via Power Marker software v3.25 (Liu and Muse, 2005). The genetic diversity of the teak populations was assessed by estimating the percentage of polymorphisms (P%), the observed number of alleles (Na), the effective number of alleles (Ne), the Shannon information index (I), the observed heterozygosity (Ho), the expected heterozygosity (He) and inbreeding coefficient [F_IS_ = 1- (Ho/He)] (Wright, 1965) via GenAIEx v6.50 software (Peakall and Smouse, 2006; 2012). The unbiased expected heterozygosity (uHe) and observed allele number via rarefaction Na (rar) were re-estimated via GenAIEx and HP-Rare 1.1 (Kalinowski, 2005), respectively, to eliminate sample size bias in the population.

### Genetic structure

The presence of subpopulations within ten teak populations was elucidated through model-based Bayesian clustering of Pritchard et al. (2000) implemented in Structure software v2.3.4 (Pritchard et al., 2000; Falush et al., 2003). A total of 5 iterations with K values ranging from 1 to 12 were run for an admixture model with correlated allele frequency. The run length was set to 1,000,000 Markov chain Monte Carlo replicates following a burn-in period of 100,000. The structure output was analysed in a structure harvester (Evanno et al., 2005) to infer the true number of K or cryptic populations.

### Genetic distance and phylogeny

Genetic distance was estimated through pair-wise population matrices of Nei’s unbiased genetic distance (Nei et al., 1983) in GenAIEx v6.50 (Peakall and Smouse, 2006; 2012). The same genetic distance matrix was used to perform principal coordinate analysis (PCoA) in GenAIEx v6.50 (Peakall and Smouse, 2006; 2012). Additionally, a population-based phylogenetic tree was constructed via the neighbor‒joining model with a bootstrap value of 1,000 in Popgene v1.32 (Yeh et al., 1997).

### Genetic differentiation at the hierarchy

The proportion of genetic differentiation and genetic isolation was further quantified via analysis of molecular variance (AMOVA) at different hierarchical levels via Arlequin v3.5 (Excoffier and Lischer, 2010).

## Results

### SNP locus information

A total of candidate gene based 98 SNPs/indels, 31 from *CAD1*, 50 from *MYB1*, and 17 from *MYB2*, were used to characterize the Indian teak populations. The minor allele frequency (MAF), a key parameter for evaluating the informativeness of bi-allelic SNP markers, ranged from 0.01 (CAD1-618G/T) to 0.50 (MYB1-1326A/G), with an average of 0.11. The gene diversity varied from 0.02 (CAD1-618G/T) to 0.50 (MYB1-1326A/G), with an average of 0.17, and the PIC value ranged between 0.02 (CAD1-618G/T) and 0.38 (MYB1-1326A/G), with an average of 0.15. Minor allele frequencies less than 5% (<0.05) are considered rare variants, whereas those above 5% (>0.05) are considered common variants. Among the 98 SNPs, 25 loci had an MAF <5% and 73 loci at MAF >5% (29 loci at MAF between 5% and 10%, 35 loci at MAF between 10% and 20%, and the remaining nine loci at MAF between 23% and 50%). Notably, nine SNPs, MYB1-1201G/A, MYB2-349delG, CAD1-349T/CA, MYB2-793C/T, MYB1-1072A/C, MYB1-1106G/C, MYB1-223G/C, MYB2-480T/A, and MYB1-1326A/G, presented the highest MAF (23%–50%), along with maximum gene diversity (0.30–0.49) and PIC values (0.26–0.38) (Supplementary Table).

### Genetic diversity

The genetic diversity of 10 teak populations was evaluated. The P% and Na were the highest in the OR population (P% = 88.78%, Na = 1.92), followed by the MH (P% = 83.67%, Na = 1.89) and KR (P% = 67.35%, Na = 1.70) populations, in that order. The lowest values were recorded in GJ (P% = 12.24%, Na = 1.12), followed by MP (P% = 20.41%, Na = 1.21). Similarly, I and He were also the highest in the OR population (I = 0.32 and He = 0.20), followed by TN (I = 0.30, He = 0.19) and KE (I = 0.27, He = 0.19), in that order. The GJ (I = 0.05, He = 0.03) and MP (I = 0.11, He = 0.07) populations presented low values for these parameters. Conversely, Ne, Ho, allelic richness estimated via rarefaction [rar (fac)] and uHe, were highest in the KE population (Ne = 1.33, Ho = 0.15, Na (rar) = 1.16, uHe = 0.20), followed by TN (Ne = 1.32, Ho = 0.15, Na (rar) = 1.16, uHe = 0.20). The lowest values for these indices were recorded in the GJ population (Ne = 1.05, Ho = 0.04, Na (rar) = 1.03, uHe = 0.04). The inbreeding coefficient (F_IS_) was highest in the UP population (0.57), followed by the OR population (0.47), and the lowest value was detected in the MP population (−0.14) (Table 2).

**TABLE 2:** Genetic diversity indices of 147 teak genotypes computed across ten Indian states.

State	Sample size	Genetic diversity indices								
		P%	Na	Ne	Na (rar)	I	Ho	He	uHe	F_IS_
All India	9	34.69	1.35	1.13	1.08	0.14	0.07	0.09	0.09	0.13
Andhra Pradesh	26	66.33	1.70	1.21	1.12	0.24	0.11	0.14	0.15	0.16
Gujarat	4	12.24	1.12	1.05	1.03	0.05	0.04	0.03	0.04	−0.10
Kerala	6	45.92	1.46	1.33	1.16	0.27	0.15	0.19	0.20	0.18
Karnataka	29	67.35	1.70	1.17	1.10	0.21	0.09	0.12	0.12	0.16
Maharashtra	24	83.67	1.89	1.22	1.12	0.26	0.11	0.15	0.16	0.28
Madhya Pradesh	4	20.41	1.21	1.12	1.07	0.11	0.08	0.07	0.09	−0.14
Odisha	26	88.78	1.92	1.29	1.16	0.32	0.09	0.20	0.20	0.47
Tamil Nadu	13	62.24	1.63	1.32	1.16	0.30	0.15	0.19	0.20	0.22
Uttar Pradesh	6	39.80	1.40	1.21	1.12	0.20	0.05	0.13	0.14	0.57
Mean	14.7	52.14	1.54	1.21	1.11	0.21	0.09	0.13	0.14	0.25

P%: percentage of polymorphism, Na: observed number of alleles, Ne: effective number of alleles, Na (rar): allelic richness calculated via rarefaction, I: Shannon’s information index, Ho: observed heterozygosity, He: expected heterozygosity and uHe: unbiased expected heterozygosity, F_IS_: inbreeding coefficient.

### Genetic structure

**FIGURE 2:**
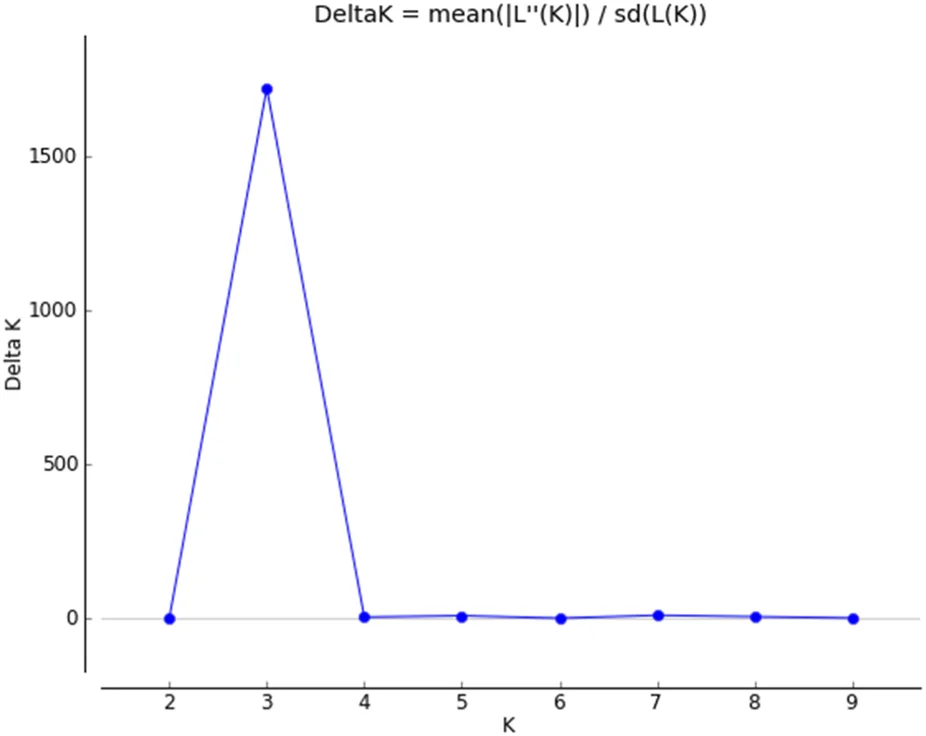
STRUCTURE analysis of 147 teak genotypes from 10 teak populations revealing three inferred subpopulations (ΔK = 3) under the admixture model with correlated allele frequencies.

**FIGURE 3:**
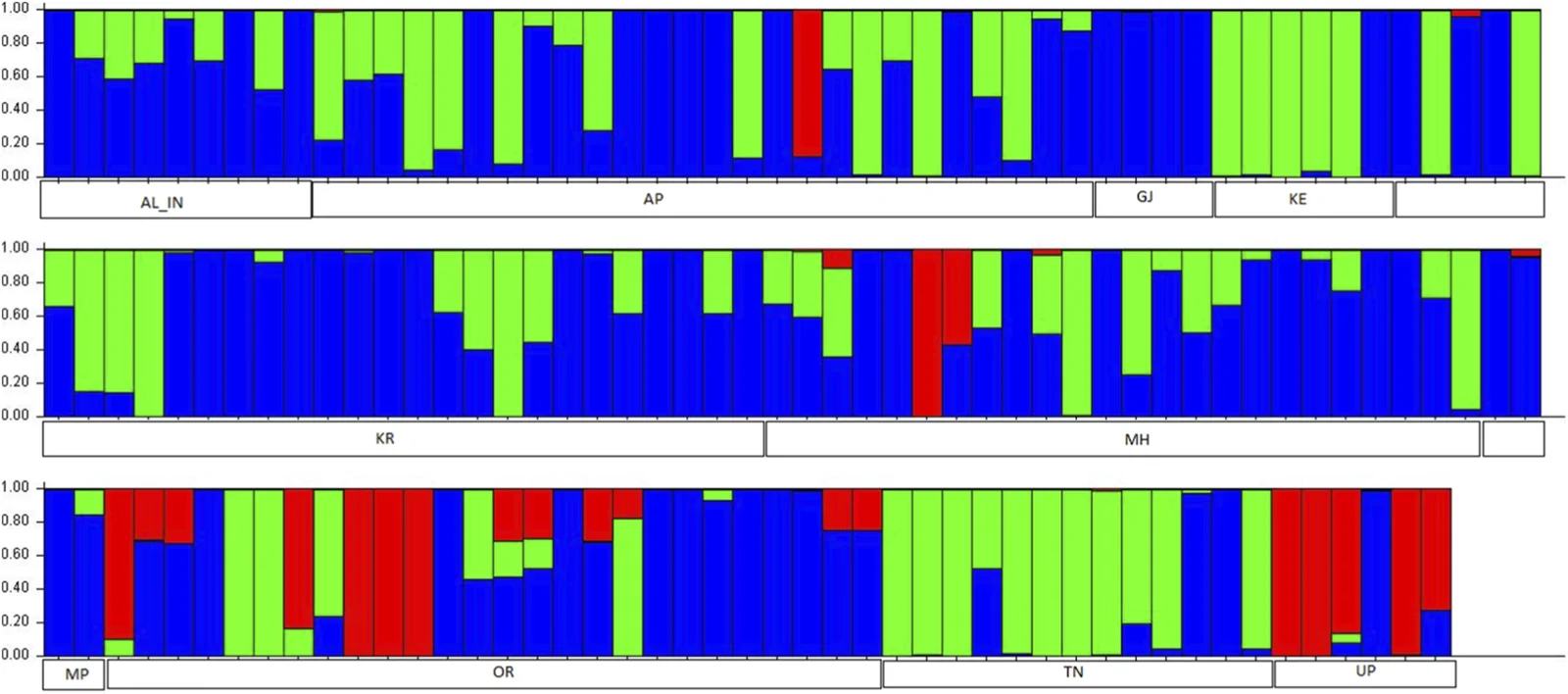
Bar plot showing the population structure of teak genotypes, with three colors indicating admixture proportions from the detected gene pools.

Structure analysis (K = 3) revealed three distinct but highly admixed subpopulations among the ten teak populations. At a membership ancestry coefficient threshold of Q > 0.7, 77 genotypes (52.38%) formed the first and largest subpopulations, predominantly comprising genotypes from GJ, MP, AP, MH, KR, AL_IN, and OR. The second subpopulation included 37 genotypes (25.17%), primarily from KE and TN. The smallest third subpopulation consisted of 12 genotypes (8.10%), mainly from UP, along with five genotypes from OR (ORPB_21, ORPB_20, ORPB_9, ORANP_3, ORANP_2), one from MH (MHAL_P4), and one from AP (APKEA_24), which exhibited a distinct allelic pattern, differentiating them from the other two subpopulations. Additionally, 21 genotypes (14.28%) presented high admixture (Q < 0.7) across all three subpopulations, mainly from AP, KR, MH, OR and TN (Figures 2, 3).

### Genetic distance and phylogeny

**FIGURE 4:**
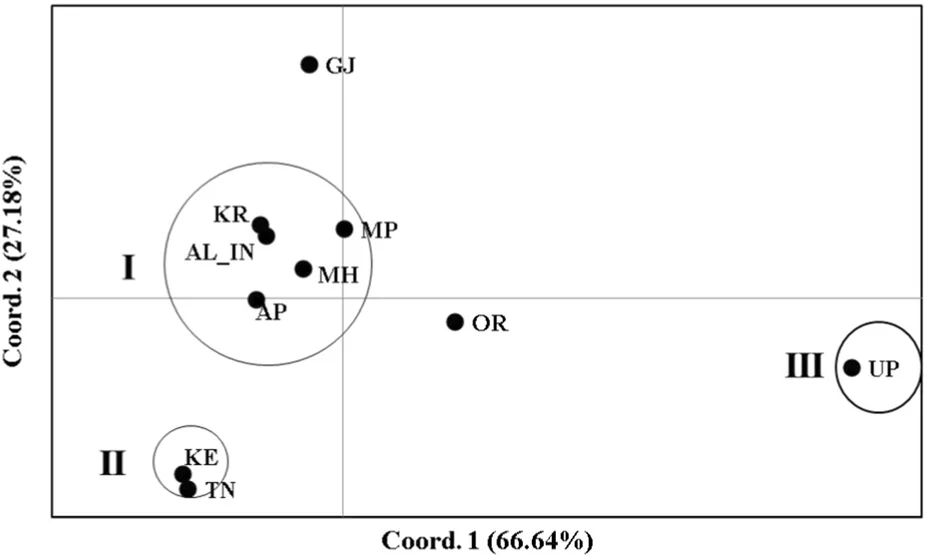
Principal coordinate analysis (PCoA) based on Nei’s genetic distance, which grouped 10 teak populations into one major and two minor clusters.

**FIGURE 5:**
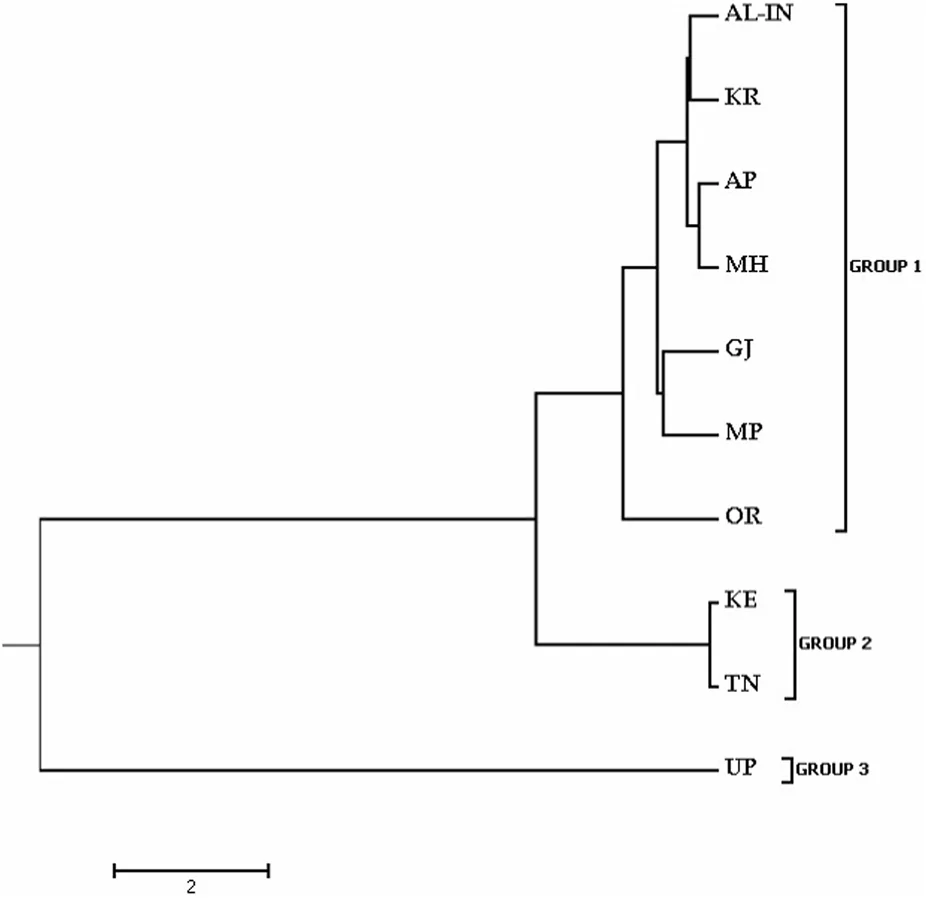
Neighbor-joining phylogenetic tree based on 1,000 bootstrap replicates grouping 10 teak populations into one major and two minor clusters.

Nei’s unbiased genetic distance matrix for the pairwise population revealed the greatest genetic distance between the UP (North India) and KE (South India) populations (0.23), whereas the lowest genetic distance was observed between the KE and TN (both from South India) populations (0) (Table 3). Principal coordinate analysis (PCoA), which is based on Nei’s unbiased genetic distance, explained 66.64% and 27.18% of the genetic variation along the first and second axes, respectively, accounting for a total of 94% of the variation, and only 6% of the variation occupied the third smallest subpopulation. PCoA clustered the UP genotypes and KE-TN genotypes distinctly and far from the large grouped genotypes of AP-KR-MP-MH-AL_IN and the highly admixed population of OR (Figure 4). The population-based phylogenetic tree aligned with the population structure analysis, where ten teak populations were grouped into three clusters. The adjacent states of GJ-MP, AP-MH, KR-AL_IN and admixed genotypes of OR comprised a single node, constituting one of the largest clusters. KE and TN formed the second cluster, and UP, the most distinct teak, created the third small cluster (Figure 5).

**TABLE 3:** Pairwise Nei’s unbiased genetic distances among teak populations from 10 Indian states.

States	AL_IN	AP	GJ	KE	KR	MH	MP	OR	TN	UP
AL_IN	0	​	​	​	​	​	​	​	​	​
AP	0.005	0	​	​	​	​	​	​	​	​
GJ	0.010	0.019	0	​	​	​	​	​	​	​
KE	0.038	0.014	0.069	0	​	​	​	​	​	​
KR	0.005	0.004	0.008	0.027	0	​	​	​	​	​
MH	0.006	0.003	0.014	0.028	0.007	0	​	​	​	​
MP	0.007	0.015	0.009	0.061	0.010	0.012	0	​	​	​
OR	0.020	0.019	0.031	0.047	0.022	0.012	0.029	0	​	​
TN	0.039	0.016	0.071	0.000	0.030	0.034	0.059	0.046	0	​
UP	0.164	0.177	0.173	0.228	0.174	0.152	0.176	0.076	0.219	0

### Genetic differentiation at the hierarchy

The analysis of molecular variance (AMOVA) revealed 85.51% genetic variation within and 14.49% genetic variation among the ten teak populations, with an Fst value of 0.14 (P < 0.001). At the level of three cryptic subpopulations and three clusters, AMOVA revealed 62.29% genetic variation within subpopulations/clusters, 35.73% genetic variation among subpopulations/clusters and 1.97% genetic variation among states within subpopulations/clusters, with an Fst value of 0.38 (P < 0.001) (Table 4).

**TABLE 4:** Genetic differentiation of 147 teak genotypes within and among states, groups and clusters.

Source of variation	Degree of freedom	sum of squares	Variance components	Variation	F_ST_
Among ten states of collection					
Among state	9	354.80	1.16	14.49	0.14 (P < 0.001)
within state	284	1952.21	6.88	85.51	
Total	293	2,307.01	8.04	100.00	
Among three subpopulations and three clusters obtained in prichard analysis and phylogenetic tree					
Among sub-populations/cluster	2	417.88	3.96	35.73	0.38 (P < 0.001)
among states within sub-population/cluster	7	100.49	0.23	1.97	
within sub-population/cluster	284	1872.14	6.89	62.29	
Total	293	2,390.51	11.07	100.00	

## Discussion

Genetic diversity, genetic differentiation, and population structure are crucial for identifying genetically rich and conservation-priority populations. The illegal felling, increasing timber demand, urbanization, natural selection, and climate change are gradually eroding existing teak genetic resources. The comprehensive assessment and conservation of the teak gene pool are of paramount importance to ensure sustainable utilization and maintain adaptive potential under future environmental challenges. In this context, molecular markers have become valuable tools for identifying genetically diverse populations. Among them, SNP markers, owing to their low mutation rate and genome-wide distribution, provide reliable inference of population differentiation and deep ancestral relationship in long-lived tree species.

### SNP locus information

Minor allele frequency, gene diversity, and PIC are key indicators of a molecular marker’s ability to differentiate individuals or populations (Botstein et al., 1980). A higher MAF generally corresponds to greater gene diversity and PIC. SNPs with MAFs less than 5% are considered low polymorphic, 5%–20% moderately polymorphic, and >20% are highly polymorphic (Gorlov et al., 2008). Among 98 SNPs/Indels studied, 25% were low, 66% were moderate, and 9% were highly polymorphic. Notably, moderate to high polymorphic SNPs are most suitable for assessing genetic diversity and population structure. Comparatively low MAF (10.6%) and PIC (0.15) values were recorded compared to those of previously reported MAF (19%) and PIC (0.22) in teak (Patturaj et al., 2025) and in *Populus tremula* (PIC = 0.55) (Kim et al., 2018). The observed disparities in the MAF and PIC values in the present investigation are attributed to the SNPs investigated in the conserved region rather than in the wider genome. Furthermore, the SNPs generated through re-sequencing of a gene and TF lead to the production of many rare variants, and all those variants were incorporated into the analysis. On the other hand, lakhs of SNPs generated through whole-genome re-sequencing filtered rare variants (MAF = 5%–10%), resulting in high MAFs and PICs. The gene diversity in this study (0.02 and 0.50) is very close to the gene diversity obtained from 3 cpSNPs (0–0.57) in the same species (Win et al., 2015a); however, the value is greater than that estimated in *Populus tremuloides* (0.0006–0.0126) (Kelleher et al., 2012). The high gene diversity in teak may be attributed to its reproductive and mating system, which confers obligate cross-pollination to the species.

### Genetic diversity

The P%, I, and Na were the highest in the OR population and the lowest in the GJ population. The P% represents the proportion of polymorphic loci, and Na represents the total number of distinct alleles observed in a population. The parameters P% and Na are strongly influenced by sample size; hence, the values could not be directly compared across populations with varying sample sizes. Furthermore, these parameters exhibit constraints in accounting for the rare allele frequency and balanced allele frequency that contribute to heterozygosity. In the investigation, a strong positive correlation existed between sample size and P% (r = 0.87) and Na (r = 0.89), suggesting that large sample sizes are more likely to capture a broader range of genetic variation due to increased chances of detecting rare alleles. Notably, when similar population sizes of OR and AP (26 genotypes each) were compared, the OR exhibited greater values for these indices, indicating greater allelic richness in the OR population. The finding is consistent with the recent studies (Hansen et al., 2015; Mohammad et al., 2022; Vaishnav et al., 2014).

Notably, the ambiguity arising from differences in sample size was addressed by calculating (Na (rar)) and uHe. Interestingly, the diversity indices Ho, uHe, Ne, and Na (rar) were observed to be highest in the KE population. These indices may provide a more robust representation of the true genetic diversity. Ho represents the proportion of heterozygotes observed at a locus in a population. The Ne provides a more informative measure of genetic diversity that accounts for rare and balanced allele frequency. The Ne precisely reflects the Ho, and its high value corresponds to high Ho. Several previous studies using co-dominant and dominant markers have also reported relatively high genetic diversity in the KE population (Nicodemus et al., 2005; Balasundaran et al., 2010; Sreekanth et al., 2012). However, the genome wide SNP diversity conducted in NTGB (Patturaj et al., 2025) did not allow direct comparison among states, as diversity was estimated in the inferred subpopulation. Moreover, a diversity study conducted in Indonesia reported higher genetic diversity in Central India and Godavari populations (Onuma et al., 2025). In the investigation, the relatively high genetic diversity in the KE population may be attributed to its humid tropical climate with moderate to high rainfall (200–500 cm), which supports semi evergreen and moist deciduous forest. The state includes parts of the Western Ghats; a biodiversity hotspot provides favourable conditions for teak growth and natural regeneration.

### Population structure

The structure analysis inferred three subpopulations among ten teak populations. The first subpopulation covers all naturally distributed regions of the Central-South-East-West Indian states of MP-GJ-AP-MH-KR-OR-AL_IN. The second subpopulation is small and consists mainly of southern peninsular states of KE-TN. These two subpopulations together explain 94% of the total genetic variation. Although these two subpopulations are genetically distinct, 21% of the KR, 35% of the AP, 13% of the MH and 15% of the OR teak genotypes from first subpopulation exhibited genetic similarity with the second subpopulation (KE-TN), suggesting substantial gene flow among adjacent populations. However, the teak from the central Indian MP and GJ appears to be relatively unique, with no genetic admixture, and possibly may reflect different origin points. Interestingly, the SNP markers revealed a previously undetected third subpopulation that mainly belonged to UP and OR. The third subpopulation is very small, with only 12 teak genotypes (8.1%); UP (UP-1, UP-D, UP-E, UP-F, and UP-N; planted in 1982–1983), along with five genotypes from OR (ORPB_21, ORPB_20, ORPB_9, ORANP_3, ORANP_2; planted in 1979), one each from MH (MHAL_P4) and AP (APKEA_24), accounted for 6% of the total genetic variation.

Previous studies (Verhaegen et al., 2010; Hansen et al., 2015) have suggested that the highly diverse East Indian teak of OR is not only distinct from the southern and central Indian teak but also a potential centre of diversity for teak in India. However, the present investigation revealed that the OR population presented genotypes associated with all three gene pools. Approximately 54% of OR teak shares genetic similarity with the first subpopulation, 15% aligns with the second sub-population, and 19% remain entirely distinct and form a separate third subpopulation alongside UP teak. Since most of the OR teak (69%) is of Indian origin, the third cryptic subpopulation of few genotypes of OR and UP is hypothesized to be exotic and not of Indian origin. The distinct genetic composition of the third subpopulation raises the possibility that it may represent an introduced lineage. One plausible hypothesis is that some of these genotypes originated from historical introductions from neighbouring regions, particularly Myanmar. Our findings are consistent with the genome wide SNP analysis of Patturaj et al. (2025), which also suggested a possible Myanmar origin for a subset of OR teak. Nevertheless, both studies highlight the need for direct comparison with reference population from Myanmar before definitive conclusion can be drawn. Interestingly, comparative genetic studies have also demonstrated that Myanmar teak presents higher private allele frequencies (Na = 2.04; He = 0.22) than most Indian populations do (Win et al., 2015a), which may indicate its potential role as a primary genetic source.

Previous studies have consistently reported that the OR teak population represent a highly diverse gene pool and showed genetic similarity with Indonesia, Africa, Thailand, and south Myanmar teak (Shrestha et al., 2005; Fofana et al., 2008; Verhaegen et al., 2010; Fofana et al., 2009; Hansen et al., 2015). However, the lack of information of planting material in the planted region in OR has created uncertainty about whether it represent a true center of diversity or a site of accumulated gene pool. Historical records indicates that Britishers established the Barbera teak forest in OR in 1910, may possibly with seeds imported from Myanmar, which could explain the unique genetic signature observed in a portion of the OR teak. Interestingly, similar movements of teak germplasms across Southeast Asia during the colonial era have been documented, with teak being one of the most valuable timbers for shipbuilding and railway sleepers. This historical context strengthens the possibility of ancient human-mediated translocation of teak genotypes across national borders.

Furthermore, OR and UP teak deviate from HWE and exhibit much lower observed heterozygosity (Ho) than expected heterozygosity (He) because of relatively high inbreeding, as evidenced by the high inbreeding coefficient (F_IS_ = 0.46 and 0.56). A relatively high inbreeding in the OR and UP teak genotypes presumably reflects a founder effect and genetic bottleneck, followed by genetic isolation and a lack of gene flow. These factors may associate with rapid loss of heterozygosity and convergence to homozygosity, leading to such a high F_IS_ value. A high inbreeding coefficient (F_IS_ = 0.45) has previously been reported in OR teak (Vaishnav, 2017). This pattern corresponds well with the classical genetic drift theory, which predicts greater allele frequency shifts in small, isolated populations, gradually reducing their adaptive potential and increasing the risk of genetic erosion.

### Genetic distance and phylogeny

The maximum genetic distance observed between UP and KE population may reflect their large geographical separation, physical barrier, restricted gene flow, and the possible presence of introduced plantation material in the UP population. In contrast, the negligible genetic distance between the KE and TN population that shares common geographical boundaries, may indicate extensive gene flow and possible continuity of teak stands across the region. However, zero genetic distance may likely reflect the limited resolution of the locus-specific SNP rather than the complete genetic identity of a population.

The phylogenetic tree construction follows Pritchard analysis, indicating three genetic clusters. The largest cluster builds from populations of geographically adjacent states, GJ-MP, AP-MH, KR-AL_IN, and OR. These populations are connected through a single node, reflecting a shared genetic lineage. The second cluster included populations from KE and TN, which may represent a southern genetic lineage potentially shaped by geographic isolation and region-specific selection pressures. Notably, a third cluster comprising UP teak, displays marked genetic divergence from the other two main clusters. It is likely that the unique genotypes of UP, where natural teak population is not found, and some parts of OR population may have originated from cross-border gene flow facilitated by human activities. Such admixture patterns are often associated with long-distance seed trade and plantation programs, which inadvertently reshuffle genetic materials and create hybrid genotypes with complex ancestry.

### Genetic differentiation at the hierarchy

The present investigation revealed relatively moderate genetic differentiation (Fst = 0.14) among ten teak populations, which may indicate limited but ongoing gene flow among the states. However, the locus specific candidate gene SNPs (98 SNPs) revealed substantially very high genetic differentiation (Fst = 0.38) among three inferred subpopulations compared to genome wide SNPs (493,591; Fst = 0.15). A relatively high genetic differentiation among subpopulations (35.73), comparable to the reported value in teak (39.43; Patturaj et al., 2025), indicates restricted gene flow among inferred subpopulations likely due to geographic isolation, ecological barrier and varying selection pressure. The high Fst value may be attributed to the functional and potentially adaptive nature of candidate gene SNPs, which are more likely to capture selection-driven divergence, whereas genome-wide SNPs mostly represent neutral variation and therefore may provide a lower estimate of population differentiation. In addition, a small number of loci analysed may have influenced the Fst estimates.

The present study provides important insights but is subject to certain limitations; 1) Sample sizes were uneven among states, although rarefaction-based approaches and unbiased diversity estimates were employed to reduce sampling bias, unequal representation may still influence estimates of genetic diversity, differentiation and population structure. (2) The analysis was based on 98 SNPs derived from three candidate genes (CAD1, MYB1 and MYB2). Candidate gene markers may capture locus specific patterns and potentially adaptive variation, but they may not fully represent genome wide diversity. Future studies incorporating high density genome wide SNP markers will provide a more comprehensive understanding of teak diversity and gene flow. (3) The detailed information regarding teak collections from different stands within individual states is unclear. The provenance trial of the NTGB reported by Kumar et al. (1998) indicated that the NTGB teak collection comprises genotypes from more than 30 stands representing 12 Indian states. Some of the well known stands included Hunsur (KR), Nilambur (KE), Wayanad (KE), Hoshangabad (MP), Amaravati (MH) and Angul (OR). As India is a hotspot of teak genetic diversity, a comprehensive pan Indian sampling from both natural well known forest and historical plantations, particularly from the Dandeli-Virnoli region of Western Ghats in Karnataka which is recognized as one of the oldest natural teak reservoirs, would provide a complete understanding of genetic diversity pattern and facilitate robust comparisons among teak genetic resources across the country.

## Conclusion

Present study demonstrated that the candidate gene SNPs are effective at assessing genetic diversity and population structure in Indian teak. The finding suggest that the OR population contain genetic components associated with two Indian teak lineage (Southern and Central Indian teak) and a third distinct lineage hypothesized to be of Myanmar origin, which may collectively contribute to the high allelic richness observed in this population. However, the OR and UP populations exhibited relatively high inbreeding coefficient, indicating reduced heterozygosity and potential genetic isolation. Promoting gene flow through appropriate breeding strategies may help improve the genetic health of these populations. Furthermore, the KE population, representing one of the oldest natural teak reservoirs, exhibited relatively high genetic diversity may likely serves as a potential center of origin. Likewise, the MP population, which possesses distinct genetic composition coupled with negligible genetic admixture with the southern teak lineage (KE-TN), may represent another teak lineage or potential center of origin. However, this inference should be interpreted cautiously due to the relatively limited sample size from the state. Future studies incorporating more uniform sampling across MP and other underrepresented region are required to better resolve the genetic diversity patterns and evolutionary history of Indian teak. Overall, the majority of Indian teak genotypes were associated with the central teak lineage. Therefore, prioritizing the southern teak lineage (KE-TN) in plantations and breeding program may help broaden its genetic base and can ensure long-term sustainability.

## Data Availability

The datasets presented in this study can be found in online repositories. The names of the repository/repositories and accession number(s) can be found below: https://www.ebi.ac.uk/eva/, PRJEB95880.
